# Supplementary material for: Bio‐Adrenomedullin Predicts Death and Major Adverse Cardiovascular Events in Cardiac Amyloidosis: A Cross‐Continental Multicenter Study
Source: J Am Heart Assoc. 2025 Dec 3;15(1):e043736. doi: 10.1161/JAHA.125.043736 (PMC12909065; doi:10.1161/JAHA.125.043736)
Supplement: Supplementary file 1 — Data S1–S2: Supplemental Methods Tables S1–S3 Figure S1 [file JAH3-15-e043736-s001.pdf]

Supplemental Material

Supplementary Methods S1 – Summary of Study Design Kumamoto University

A total of 57 consecutive patients diagnosed with ATTR-CA at Kumamoto University Hospital between February 2018 and November 2020 were enrolled. All of the patients were diagnosed with ATTR-CA based on either non-invasive diagnostic criteria, using 99mTc-labelled pyrophosphate scintigraphy and assessment of M-protein, or invasive (biopsy) criteria as per the current Japanese guideline. (25) Obtained plasma samples were kept frozen at −80°C until used in the ADMYLO analysis.

Supplementary Methods S2 – Summary of Study Design Washington University

The ACE-MOVE Registry ("prospective charACterization of patiEnts with AMyloidosis focusing on diagnOsis and innoVative trEatment to improve outcomes Registry") is a single-centre prospective registry that collects blood samples, data, surveys, and 6-minute walk tests from patients with amyloidosis at study baseline, 6 months and 1, 2, and 3 years after baseline. If the patient was not returning for a clinical visit, follow up was completed remotely and through record review. The study was approved by the Washington University Institutional Review Board and carried out in compliance with the Declaration of Helsinki. Patients with a confirmed or suspected diagnosis of ATTR, AL, AA, or other amyloidosis who were $\geq$18 years of age at time of consent were eligible for enrolment. Patients who had a known infection with Hepatitis B or C, HTLV, or HIV were excluded. All patients provided informed consent, which includes allowance for future research use of data and samples, prior to inclusion in the ACE-MOVE Registry. All blood samples and data used in the ADMYLO analysis were collected between Feb 2019 and June 2023.

Baseline Characteristics of Observation vs. Validation Cohort

Table S1: Summary of baseline characteristics of 210 patients with cardiac transthyretin amyloidosis (ATTR-CA; n=160), cardiac immunoglobulin light chain amyloidosis (AL-CA; n=49) or cardiac serum amyloid A amyloidosis (AA-CA; n=1). Baseline characteristics are compared between the observation cohort (n=86) and the validation cohort (n=124). Continuous and categorical data are presented as median [interquartile range] and count (percentage), respectively. P-values indicating statistically significant differences between the observation and validation cohort (p<0.05) are highlighted in bold font.

Abbreviations not spelled out above: Bio-ADM = Bioactive adrenomedullin; eGFR = estimated glomerular filtration rate; FLC-Diff = Difference in free immunoglobulin light chains; hs-cTnT = high-sensitivity cardiac troponin T; IVSd = interventricular septal thickness end-diastole; LVEF = left ventricular ejection fraction; LVPWd = left ventricular posterior wall thickness end-diastole; MACE = major adverse cardiac events; MV DT = mitral valve deceleration time; NAC = National Amyloidosis Centre; NT-proBNP = N-terminal pro B-type natriuretic peptide.

Variable	n	Entire Cohort (n=210)	Observation Cohort (n=86)	Validation Cohort (n=124)	p-value
Bio-ADM (pg/mL)	210	18.8 [18.8-26.4]	19.4 [18.8-28.8]	18.8 [18.8-23]	0.069
Age (years)	210	77 [70-81]	80 [75-83]	73 [68-78.3]	<0.001
Sex (male)	210	173 (82.4)	68 (79.1)	105 (84.7)	0.387
Body Mass Index (kg/m2)	196	24.9 [22.6-27.9]	24.6 [22.5-26.8]	25.3 [22.6-29.4]	0.088
Systolic Blood Pressure (mmHg)	136	118 [106-133.3]	115.5 [103-124]	120.5 [108.5-137]	0.033
Diastolic Blood Pressure (mmHg)	136	71 [64-79]	66 [60.8-75.8]	72.5 [65-79]	0.156
Type of Cardiac Amyloidosis	210				0.007
ATTR-CA (wild-type)		138 (65.7)	66 (76.7)	72 (58.1)	
ATTR-CA (variant)		21 (10)	2 (2.3)	19 (15.3)	
ATTR-CA (not genotyped)		1 (0.5)	1 (1.2)	0 (0)	
AL-CA		49 (23.3)	17 (19.8)	32 (25.8)	
AA-CA		1 (0.5)	0 (0)	1 (0.8)	
Disease-modifying Therapy at Baseline (yes)	175	52 (29.7)	20 (23.3)	32 (36)	0.095
Diagnosed Chronic Kidney Disease (yes)	210	88 (41.9)	31 (36)	57 (46)	0.197
Diagnosed Coronary Artery Disease (yes)	206	54 (26.2)	31 (36)	23 (19.2)	0.011
Atrial Fibrillation (yes)	164	40 (24.4)	17 (22.4)	23 (26.1)	0.705
Conduction Blocks (yes)	158	69 (43.7)	44 (58.7)	25 (30.1)	<0.001
LVEF (%)	173	55.9 [48-63]	57.5 [50-65]	55 [46-62.2]	0.111
IVSd (mm)	150	16 [14-18.3]	17 [15-19]	15.6 [13.4-17.6]	0.005
LVPWd (mm)	147	15 [13.3-18]	15 [13.8-17.3]	15.6 [13.3-18]	0.754
MV DT (ms)	86	166.5 [139-211.8]	168 [139-222]	166 [141-203]	0.809
E/A Ratio	93	1.3 [0.8-2.9]	1.5 [1.1-2.9]	1.2 [0.7-2.9]	0.160

NT-proBNP (ng/L)	201	1449.1 [482-3837]	2741 [1330-6438]	724 [321.4-2154]	<0.001
Hs-cTnT (ng/L)	194	45.7 [30-76]	52 [34-77.8]	40.9 [29-64.2]	0.028
Creatinine (mg/dL)	210	1.1 [0.9-1.4]	1.2 [1-1.4]	1.1 [0.9-1.4]	0.054
eGFR (ml/min/1.73m2)	209	55 [43-69]	55 [42.3-68.8]	55.5 [43.5-69.5]	0.951
Urea (g/L)	140	36.2 [21.6-52.3]	49 [38-63]	19.2 [16.6-23.8]	<0.001
FLC-diff (mg/L)	40	36.2 [5.9-301]	104.3 [23.7-445.9]	25.5 [5.1-280.1]	0.176
NAC Staging System (ATTR-CA)	154				0.002
NAC I		97 (63)	35 (51.5)	62 (72.1)	
NAC II		38 (24.7)	18 (26.5)	20 (23.3)	
NAC III		19 (12.3)	15 (22.1)	4 (4.7)	
MayoATTR Staging System (ATTR-CA. wild-type)	122				0.004
MayoATTR I		74 (60.7)	31 (50.8)	43 (70.5)	
MayoATTR II		32 (26.2)	16 (26.2)	16 (26.2)	
MayoATTR III		16 (13.1)	14 (23)	2 (3.3)	
Mayo2004 Staging System (AL-CA)	46				0.020
Mayo2004 I		7 (15.2)	0 (0)	7 (24.1)	
Mayo2004 II		15 (32.6)	4 (23.5)	11 (37.9)	
Mayo2004 III		24 (52.2)	13 (76.5)	11 (37.9)	
Mayo2012 Staging System (AL-CA)	37				0.078
Mayo2012 I		6 (16.2)	1 (6.2)	5 (23.8)	
Mayo2012 II		10 (27)	3 (18.8)	7 (33.3)	
Mayo2012 III		12 (32.4)	5 (31.2)	7 (33.3)	
Mayo2012 IIII		9 (24.3)	7 (43.8)	2 (9.5)	
Death within 2 Years (yes)	210	30 (14.3)	16 (18.6)	14 (11.3)	0.197
MACE within 2 Years (yes)	210	98 (46.7)	34 (39.5)	64 (51.6)	0.113

Baseline Characteristics of ATTR-CA vs. AL-CA Patients

Table S2: Summary of baseline characteristics of 209 patients with cardiac transthyretin amyloidosis (ATTR-CA; n=160) or cardiac immunoglobulin light chain amyloidosis (AL-CA; n=49). Baseline characteristics are compared between AL-CA (n=49) and ATTR-CA (n=160) patients. Continuous and categorical data are presented as median [interquartile range] and count (percentage), respectively. P-values indicating statistically significant differences between the AL-CA and ATTR-CA subgroups (p<0.05) are highlighted in bold font.

Abbreviations not spelled out above: Bio-ADM = Bioactive Adrenomedullin; eGFR = estimated glomerular filtration rate; hs-cTnT = high-sensitivity cardiac troponin T; IVSd = interventricular septal thickness end-diastole; LVEF = left ventricular

ejection fraction; LVPWd = left ventricular posterior wall thickness end-diastole; MACE = major adverse cardiac events; MV DT = mitral valve deceleration time; NT-proBNP = N-terminal pro B-type natriuretic peptide.

Variable	n	Combined (n=209)	AL-CA (n=49)	ATTR-CA (n=160)	P-value
Bio-ADM (pg/mL)	209	18.8 [18.8-25.9]	18.8 [18.8-31.1]	18.8 [18.8-25.7]	0.755
Age (years)	209	77 [70-81]	68 [61-77]	78 [72-82]	<0.001
Sex (male)	209	172 (82.3)	40 (81.6)	132 (82.5)	1.000
Body Mass Index (kg/m2)	195	24.9 [22.6-27.8]	26 [23.6-30.6]	24.7 [22.4-27.4]	0.046
Systolic Blood Pressure (mmHg)	135	118 [106.5-133.5]	120 [104-137]	118 [109-132.3]	0.940
Diastolic Blood Pressure (mmHg)	135	71 [64-79]	73 [64.5-77.5]	71 [64-80]	0.860
Disease-modifying Therapy at Baseline (yes)	175	52 (29.7)	23 (79.3)	29 (19.9)	<0.001
Diagnosed Chronic Kidney Disease (yes)	209	87 (41.6)	19 (38.8)	68 (42.5)	0.766
Diagnosed Coronary Artery Disease (yes)	205	53 (25.9)	15 (30.6)	38 (24.4)	0.493
Atrial Fibrillation (yes)	164	40 (24.4)	5 (17.9)	35 (25.7)	0.521
Conduction Blocks (yes)	158	69 (43.7)	9 (36)	60 (45.1)	0.533
LVEF (%)	172	55.8 [47.7-63]	55.5 [47.5-63.5]	55.8 [47.7-62.9]	0.994
IVSd (mm)	149	16 [14-18.1]	16 [12.5-18]	16 [14.1-18.3]	0.216
LVPWd (mm)	146	15 [13.4-18]	14 [11-17]	15.6 [13.7-18]	0.038
MV DT (ms)	86	166.5 [139-211.8]	149 [115-159]	171 [140.5-212]	0.143
E/A Ratio	92	1.3 [0.8-2.9]	1.7 [1-3]	1.3 [0.8-2.8]	0.513
NT-proBNP (ng/L)	201	1449.1 [482-3837]	2553 [713-8105.5]	1191.1 [442.8-2913]	<0.001
Hs-cTnT (ng/L)	193	45.7 [30-76]	44.5 [25.3-90.8]	45.7 [30.1-70]	0.925
Creatinine (mg/dL)	209	1.1 [0.9-1.4]	1.3 [1.1-2.1]	1.1 [0.9-1.3]	<0.001
eGFR (ml/min/1.73m2)	208	55 [43-69.3]	53.4 [31.7-66.3]	56 [45-70]	0.175
Urea (g/L)	140	36.2 [21.6-52.3]	47 [40-97]	35 [20.4-49.5]	<0.001
Death within two Years (yes)	209	30 (14.4)	12 (24.5)	18 (11.2)	0.038
MACE within two Years (yes)	209	97 (46.4)	31 (63.3)	66 (41.2)	0.011

Bio-ADM as part of Staging Systems for AL-CA

Table S3: Bioactive adrenomedullin (bio-ADM) as part of validated prognostic staging systems for the endpoint all-cause death, including the Mayo2004 and Mayo2012 staging systems for cardiac immunoglobulin light chain amyloidosis (AL-CA) patients.

The overall accuracy of the original staging system and the extended staging system, incorporating categorized bio-ADM (> 29 pg/mL), was assessed by use of the concordance index (C-index). The prognostic performance of individual components of each extended staging system (i.e., original + bio-ADM) was assessed in multivariable Cox regression models. Results are presented as hazard ratios (HR) with 95% confidence intervals (95% CI). The likelihood ratio chi-squared (LR $\chi2$) was used to estimate the contribution of each covariate to the overall model. P-values indicating statistical significance (i.e., $p<0.05$) are highlighted in bold font. Abbreviations not spelled out above: FLC-Diff = Difference in free immunoglobulin light chains; hs-cTnT = high-sensitivity cardiac troponin T; NT-proBNP = N-terminal pro B-type natriuretic peptide. *Or cardiac troponin I/T values above thresholds converted according to Muchtar et al. (27)

**Staging System**	**Overall Accuracy of Staging System**			**Prognostic Performance of Individual Staging System Components in Multivariable Model**			
	**Original**<br>**C-index** (bootstrap-corrected)	**Original + bio-ADM**<br>**C-index** (bootstrap-corrected)	**P-value** (added value)	**Component**	**HR (95% CI)**	**LR $\chi2$**	**P-value**
**Mayo2004** (AL-CA; n=46)	0.599	0.622	0.187	NT-proBNP (≥332 ng/L)	955.0 (0.0 – 1.17^{26})	0.1	0.800
				hs-cTnT* (≥50 ng/L)	1.74 (0.46 – 6.59)	0.7	0.413
				Bio-ADM (>29 pg/mL)	2.27 (0.69 – 7.46)	1.8	0.176
**Mayo2012** (AL-CA; n=37)	0.727	0.721	0.210	NT-proBNP (≥1800 ng/L)	3.63 (0.37 – 35.5)	1.2	0.267
				hs-cTnT* (>40 ng/L)	1.17 (0.22 – 6.37)	0.0	0.852
				FLC-Diff (≥180 mg/L)	4.97 (1.09 – 22.7)	4.3	**0.038**
				Bio-ADM (>29 pg/mL)	2.87 (0.56 – 14.7)	1.6	0.208

Correlation Analysis

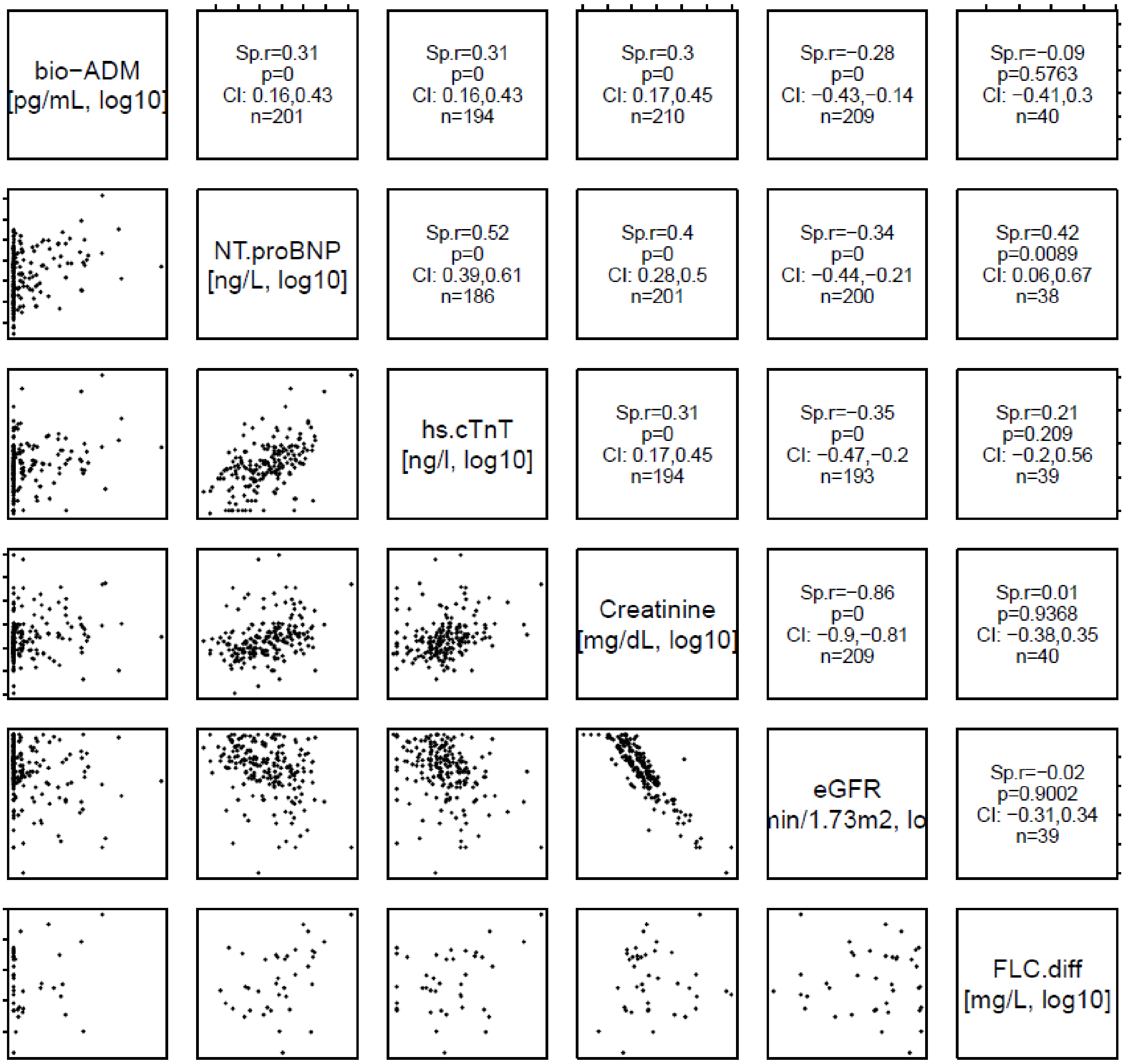

<u>Figure S1:</u> Summary of results from pairwise Spearman's rank correlation analyses between bioactive Adrenomedullin (bio-ADM), N-terminal pro B-type natriuretic peptide (NT-proBNP), high-sensitivity cardiac troponin T (hs-cTnT), creatinine, estimated glomerular filtration rate (eGFR), and the difference in free immunoglobulin light chains (FLC-Diff, only for patients with cardiac immunoglobulin light chain amyloidosis). The Spearman's rank correlation coefficient r (Sp.r) is reported with corresponding 95% confidence interval (CI) and p-value (p).

In descending order, bio-ADM correlated (all p<0.001) with NT-proBNP (r=0.31, 95% CI 0.19–0.41), hs-cTnT (r=0.31, 95% CI 0.18–0.42), creatinine (r=0.30, 95% CI 0.16–0.42), and eGFR (r=-0.28, 95% CI -0.41 – -0.16). No correlation of bio-ADM with FLC-diff was found in AL-CA patients (r=-0.09, 95% CI -0.41–0.3; p=0.576). Notably, the correlation of bio-ADM with eGFR was weaker than that of NT-proBNP (r=-0.34, 95% CI -0.47 – -0.22; p<0.001) and hs-cTnT (r=-0.35, 95% CI -0.49 – -0.21; p<0.001) with eGFR.
